# Supplementary material for: Conditional chemoconnectomics (cCCTomics) as a strategy for efficient and conditional targeting of chemical transmission
Source: eLife. 2024 Apr 30;12:RP91927. doi: 10.7554/eLife.91927 (PMC11060718; doi:10.7554/eLife.91927)
Supplement: Figure 3—source data 3. [file elife-91927-fig3-data3.zip › Figure 3 source data3 part 2.zip!/Figure 3-Source data 3-part2/1060-9-CRISPResso_Report_q0_g4o36.zip!/CRISPResso_on_1060_12/1a.Read_barplot.pdf]

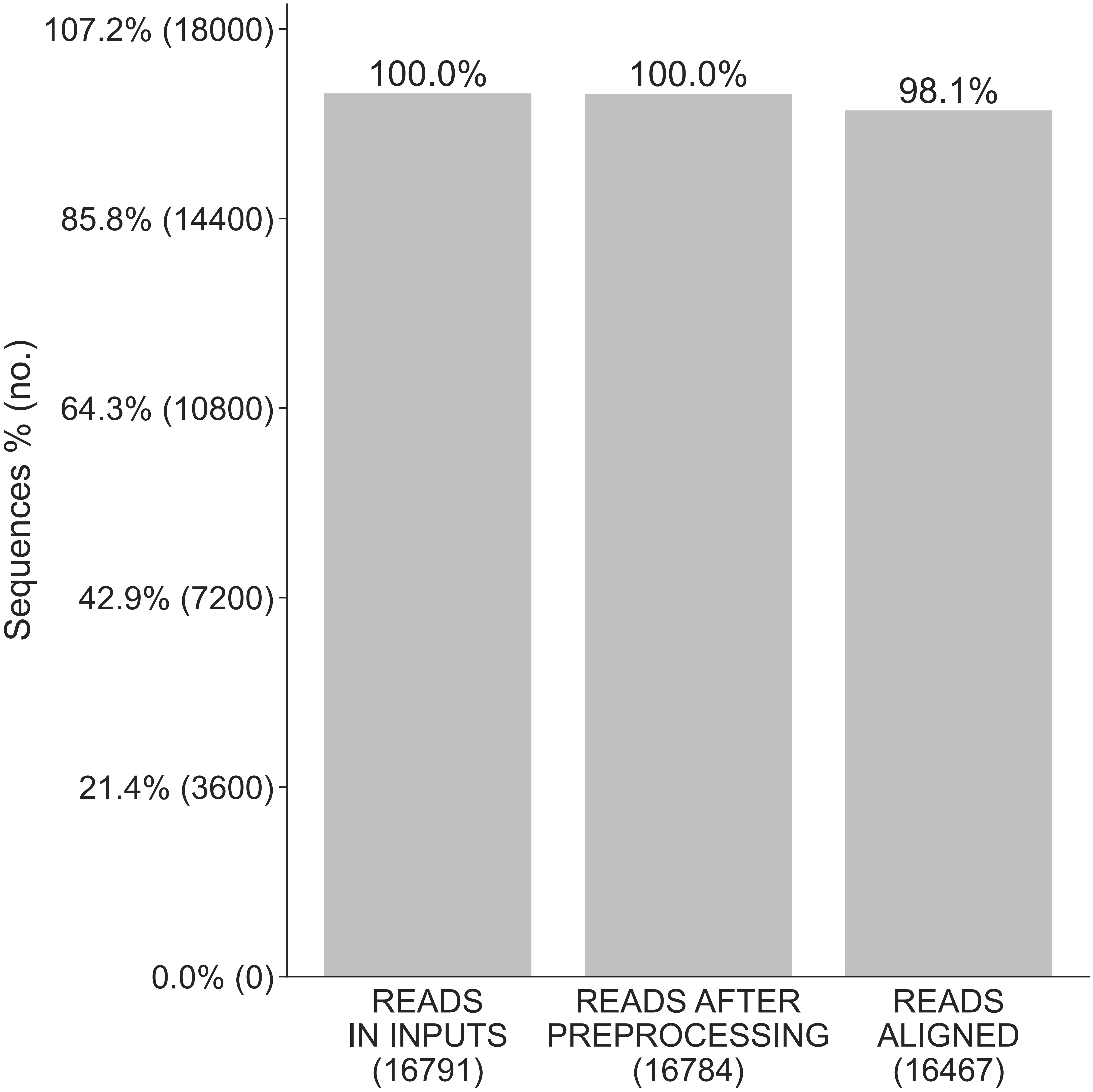

Sequences % (no.)
107.2% (18000)
85.8% (14400)
64.3% (10800)
42.9% (7200)
21.4% (3600)
0.0% (0)
100.0%
100.0%
98.1%
READS IN INPUTS (16791)
READS AFTER PREPROCESSING (16784)
READS ALIGNED (16467)
